# Regression of experimental NIS-expressing breast cancer brain metastases in response to radioiodide/gemcitabine dual therapy

**DOI:** 10.18632/oncotarget.10238

**Published:** 2016-06-23

**Authors:** Corinne Renier, John Do, Andrea Reyna-Neyra, Deshka Foster, Abhijit De, Hannes Vogel, Stefanie S. Jeffrey, Victor Tse, Nancy Carrasco, Irene Wapnir

**Affiliations:** ^1^ Department of Surgery, Stanford University School of Medicine, Stanford, CA, USA; ^2^ Department of Cellular and Molecular Physiology, Yale University, New Haven, CT, USA; ^3^ Department of Radiology and Molecular Imaging Program Stanford, Stanford, CA, USA; ^4^ Molecular Functional Imaging Lab, ACTREC Tata Memorial Centre, Navi Mumbai, India; ^5^ Department of Pathology, Stanford University School of Medicine, Stanford, CA, USA; ^6^ Department of Neurosurgery, Stanford University School of Medicine, Stanford, CA, USA

**Keywords:** sodium/iodide symporter (NIS), radioiodide therapy, breast cancer brain metastases (BCBMs)

## Abstract

Treating breast cancer brain metastases (BCBMs) is challenging. Na^+^/I^−^ symporter (NIS) expression in BCBMs would permit their selective targeting with radioiodide (^131^I^−^). We show impressive enhancement of tumor response by combining^131^I^−^ with gemcitabine (GEM), a cytotoxic radiosensitizer. Nude mice mammary fat-pad (MFP) tumors and BCBMs were generated with braintropic MDA-MB-231Br cells transduced with bicistronically-linked NIS and firefly luciferase cDNAs. Response was monitored in vivo via bioluminescent imaging and NIS tumor expression.^131^I^−^/GEM therapy inhibited MFP tumor growth more effectively than either agent alone. BCBMs were treated with: high or low-dose GEM (58 or 14.5 mg/Kg×4); ^131^I^−^ (1mCi or 2×0.5 mCi 7 days apart); and ^131^I^−^/GEM therapy. By post-injection day (PID) 25, 82-86% of controls and 78-83% of ^131^I^−^-treated BCBM grew, whereas 17% low-dose and 36% high-dose GEM regressed. The latter tumors were smaller than the controls with comparable NIS expression (~20% of cells). High and low-dose ^131^I^−^/GEM combinations caused 89% and 57% tumor regression, respectively. High-dose GEM/^131^I^−^ delayed tumor growth: tumors increased 5-fold in size by PID45 (controls by PID18). Although fewer than 25% of cells expressed NIS, GEM/^131^I^−^ caused dramatic tumor regression in NIS-transduced BCBMs. This effect was synergistic, and supports the hypothesis that GEM radiosensitizes cells to ^131^I^−^.

## INTRODUCTION

Breast cancer brain metastases (BCBMs) are challenging to treat, and the prognosis for affected patients is poor relative to that of patients with metastases elsewhere [1, 2]. Indeed, central nervous system (CNS) involvement occurs in a significant proportion of patients with triple negative [TN, (estrogen receptor, progesterone receptor, and HER2-negative)] and HER2+ metastatic subtypes. In general, systemic therapies are less successful in the CNS than at other metastatic sites. The blood-brain barrier (BBB) has been thought to impair the entry of cytotoxic, hormonal, and biological agents, providing one explanation for their limited activity against BCBMs. Additionally, the difficulty of achieving and sustaining optimal concentrations of drugs is likely compounded by rapid drug efflux [3]. Thus, for patients with BCBMs, surgical resection and radiation therapy continue to be the most successful therapeutic interventions, but are associated with debilitating neurological and neurocognitive deficits.

Clearly, ideal strategies would selectively target BCBMs without affecting the normal brain tissue. We measured the effectiveness of targeted internal radiotherapy with ^131^I^−^ (radioiodide) combined with a radiosensitizing agent in a preclinical BCBM mouse model. ^131^I^−^ therapy is worth testing in BCBMs because the endogenous mechanism of Na^+^/I^−^ symporter (NIS)–mediated iodide (I^−^) transport is present in many breast cancer cells [4]. NIS is an intrinsic plasma membrane glycoprotein that mediates active I^−^ transport in the thyroid and a few other tissues, including the lactating breast, salivary glands, stomach, and small intestine [5]. NIS couples the transport into the cell of one I^−^ ion, against its concentration gradient, to that of two Na^+^ ions, down their concentration gradient, which is generated by the Na^+^/K^+^ ATPase [6, 7]. NIS-driven I^−^ transport is the key first step in the biosynthesis of the thyroid hormones (T_3_ and T_4_). Clinically, the most relevant application of NIS has been its use in targeted intracellular delivery of high-energy ^131^I^−^ to ablate toxic nodules and thyroid cancer metastases. A second key clinical application of NIS has been its use in thyroid imaging, which is based on translocation of iodide radioisotopes and pertechnetate (^99m^TcO_4_^−^), another NIS substrate. Since NIS was identified and began to be characterized at the molecular level, its preclinical and clinical applications have expanded greatly: it is now used as both a reporter and a therapeutic molecule [5].

I^−^ concentrated in maternal milk is the only I^−^ available to the nursing newborn for use in the biosynthesis of his or her thyroid hormones, T_3_ and T_4_. The accumulation of I^−^ in the milk is made possible by the functional expression of NIS in breast cells during lactation [8, 9]. This observation led us to discover that NIS is also expressed in breast cancer. NIS is endogenously expressed to varying degrees in over 80% of breast cancers and, interestingly, in over 50% of the TN BCBMs studied by our group [4, 10]. Focal radioiodide uptake has been demonstrated in imaging studies of patients with NIS-expressing locally advanced or metastatic breast cancers [11, 12].

Exogenous NIS gene transfer into non-thyroid tumor cells has been carried out successfully in multiple studies reporting selective killing of these cells by NIS-mediated ^131^I^−^ accumulation [5, 13, 14]. The effectiveness of targeted ^131^I^−^ therapy has been shown in prostate cancer, multiple myeloma, breast cancer and intracerebral glioma xenograft models [14–16].

In breast cancer, where NIS is often expressed endogenously, the radioablative effects of NIS-mediated ^131^I^−^ accumulation could conceivably be enhanced by combining ^131^I^−^ with an anticancer agent that increases tumor cell vulnerability to the DNA-damaging effects of radiation. Gemcitabine (GEM) is a deoxycytidine analogue used in the treatment of patients with metastatic breast cancer. As first-line therapy, it has reported response rates of 12-37% [17]. The anti-tumor and radiosensitizing properties of GEM have been demonstrated for example in bladder, mucoepidermoid lung, pancreatic, head and neck, colon, and breast tumor cell lines [18, 19]. Clinically, GEM has been co-administered with external beam radiation therapy for pancreas, head and neck, and bladder cancer, as well as in BCBMs [20–23]. Thus, we sought to investigate the effectiveness of concurrent GEM with NIS-mediated ^131^I^−^ therapy in an experimental BCBM mouse model, and obtained highly promising results [24].

## RESULTS

### Immunoblot analysis of transduced MDA-MB-231Br

**Figure 1:**
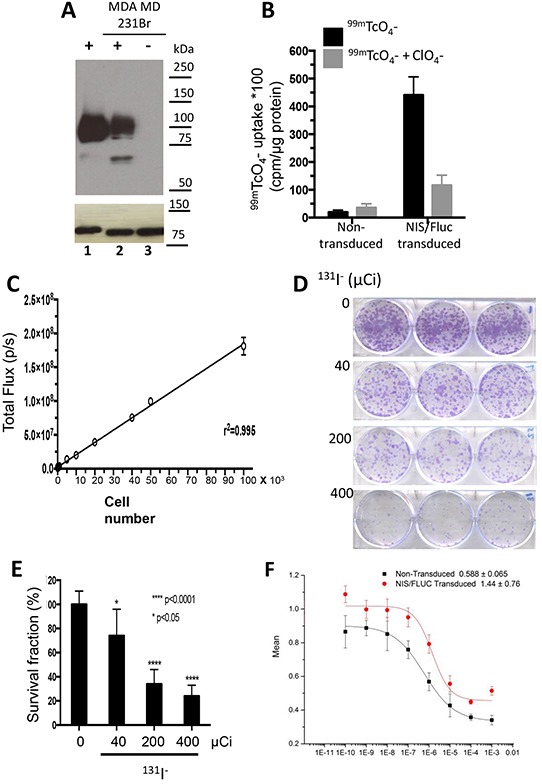
Cell characterization and cytotoxicity studies MDA-MB-231Br cells were stably transduced with a lentiviral vector containing human NIS cDNA linked via a bicistronic construct to the firefly luciferase 2 cDNA (NIS-IRES-luc2, NIL), driven by a CMV promoter. **A.** Immunoblot analysis of membrane fractions (15 μg) derived from transduced control MDCK cells, MDA-MB-231Br-NIL (lanes 1 and 2) and non-transduced MDA-MB-231Br (lane 3). Distinct bands corresponding to NIS protein are seen in lanes 1 and 2, but completely absent in 3. **B.** Functional expression of NIS was measured by quantitating ^99m^TcO_4_^−^ uptake in single-cell-derived clonal populations. **C.** Functional expression of luciferase was measured using *in vitro* bioluminescence. Cell number correlates linearly with in vitro bioluminescence (r^2^ = 0.995). **D–E.** *In vitro* cytotoxicity in MDA-MB-231Br-NIL cells exposed to increasing doses of ^131^I^−^ (0, 40, 200, or 400 μCi) for 7 hours. [D] Representative photographs of a^131^I^−^ clonogenic assay and [E] surviving fraction or colony-forming efficiency determined 10 to 14 days later (mean ± SD; * *p* < 0.05; **** *p* < 0.0001 post hoc Tukey's multiple comparisons test). **F.** Dose-dependent effect of gemcitabine (GEM) on cell survival. Cell viability determined by MTS assay after a 48-hour incubation of 0.0001 to 1000μM GEM relative to HBSS-treated control. The concentration of the drug yielding half-maximal response (EC50) was estimated to be 1.44 ± 0.76 μM for the transduced cells and 0.588 ± 0.065 μM for the parental cells.

NIS expression was demonstrated in MDCK and MDA-MB-231Br cells that were stably transduced with a lentiviral vector containing NIS cDNA linked via a bi-cistronic cassette to firefly luciferase 2 (Fluc2) cDNA, under the CMV promoter (NIS-IRES-LUC=NIL). Two bands corresponding to NIS were detected in the transduced cells, in contrast to the complete absence of expression in the non-transduced MDA-MB-231Br cells [Figure 1A].

### NIS-mediated ^131^I^−^ accumulation ablates MDA-MB-231Br cells in a dose-dependent manner

*In vitro* I^−^ uptake and bioluminescence assays were performed to characterize NIS and Fluc expression in transduced MDA-MB-231Br cells. The selected clone exhibited a 25-fold increase in NIS-mediated ^99m^TcO_4_^−^ uptake [Figure 1B], as compared to non-transduced cells; the *in vitro* bioluminescence (BLI) signal correlated linearly with the number of cells [Figure 1C]. Flow cytometry analysis showed that 97.5% of the transduced cells expressed NIS and Fluc.

Cell sensitivity to ^131^I^−^ was analyzed by exposing cells to various doses of this radioisotope, and performing a clonogenic survival assay. The fraction of cells that survived decreased in a dose-dependent manner [Figure 1D and 1E]. The highest dose tested (400 μCi) reduced the fraction of surviving cells to only 24%.

GEM cytotoxicity was evaluated in parental and transduced MDA-MB-231Br-NIL cells incubated for 48 hours with doses ranging from 0.0001 to 1000 μM. Following treatment, cell viability was determined with a MTS assay. GEM treatment had a dose-dependent effect on cell survival [Figure 1F]. Maximal response was reached with 1 mM, which caused a 56% decrease in cell survival. The concentration of the drug yielding half-maximal response (EC50) was estimated to be 1.44 ± 0.76 μM for the transduced cells and 0.588 ± 0.065 μM for the parental cells.

### MFPs and BCBMs actively transport ^123^I^−^

**Figure 2:**
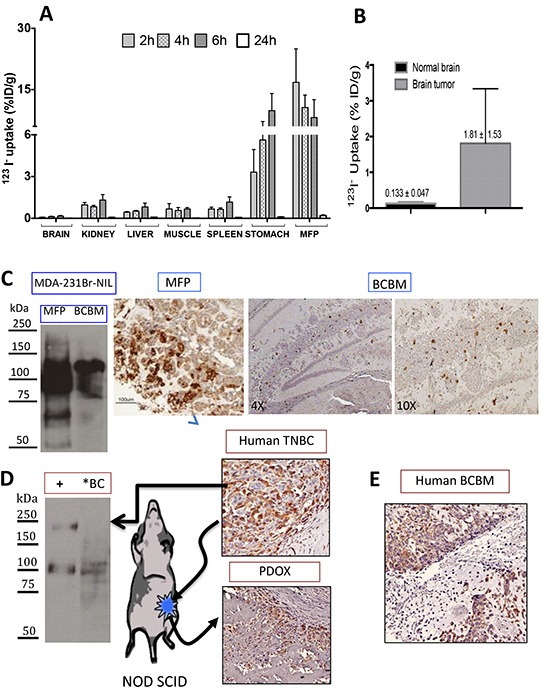
Radioiodide uptake in MDA-MB-231Br-NIL MFP and BCBM xenografts **A.** Tissue biodistribution of 40 μCi ^123^I^−^ i.v. at 2, 4, and 6 hours in mice bearing NIS-expressing MDA-MB-231Br-NIL MFP xenografts. Data represent the mean ± SD of ^123^I^−^ uptake expressed as the percentage of the injected dose per tissue or organ (% ID/g). MFP tumors show peak accumulation of 16.8 ± 8.2 %ID/g at 2 hours decreasing to about half at 6 hours and exceeding the stomach at the earlier timepoints. **B.** Radioiodide uptake in brain 1 hour post-injection of ^123^I^−^ demonstrates that brains with tumors take up 13.6 times more ^123^I^−^ than normal brain tissue (n = 3). **C.** Immunoblot of NIS expression in MFP and BCBM xenografts shows a predominant band corresponding to the mature polypeptide and a band that migrates faster, corresponding to the partially glycosylated polypeptide. Immunohistochemical analysis of NIS expression in MFP and BCBM xenografts on day 11 post-implantation. **D.** Immunoblot of NIS expression in human primary triple negative breast cancer [TNBC] (*BC), and of a protein lysate (2 μg) obtained from MDCK transfected with hNIS used as a positive control showing the same electrophoretical pattern. This same tumor was minced in RPMI tissue culture and implanted into the MFP in a NOD SCID mouse to generate a patient-derived orthotopic xenograft (PDOX). Comparable NIS immunoreactivity is observed in the human TNBC and corresponding PDOX. **E.** NIS expression was also assessed by IHC on tissue sections of a TNBC BCBM patient is shown.

*In vivo* ^123^I^−^ uptake measurements in mice with MFP tumors showed peak uptake by these tumors at 2 hours of 16.8 ± 8.2 % ID/g, decreasing to 10.6 ± 3.0 and 8.2 ± 4.4 %ID/g at 4 and 6 hours respectively. These levels exceed the stomach at the early timepoints but are comparable to the peak uptake recorded at 6 hours of 9.9 ± 4.2 %ID/g. Other organs showed much lower accumulation (1.00 %ID/g or less at all timepoints). Notably, normal brain tissue had the lowest signal of all organs studied, peaking at 6 hours of 0.16 % ID/g compared to 0.08 %ID/g at 2 hours [Figure 2A]. In experiments with stereotactic-generated BCBMs, ^123^I^−^ uptake at 1 hour was approximately 13.6 times greater in these mice (1.81 ± 1.53 %ID/g) than in control animals (0.133 ± 0.047 %ID/g) [Figure 2B].

Immunoblot analysis of tissue samples derived from MFP and BCBMs demonstrated NIS protein expression: a predominant band corresponding to the mature polypeptide and a band that migrates faster, corresponding to the partially glycosylated polypeptide [Figure 2C]. Consistent with the immunoblot, fewer cells expressing NIS were observed by IHC in BCBMs than in MFPs. Similarly, NIS expression was demonstrated in a human TN breast cancer by immunoblot and IHC [Figure 2D] as well as in a human TN BCBM [Figure 2E]. A small portion of the former was freshly implanted orthotopically into a NOD SCID mouse (PDOX). Archival tissue section of a second passage xenograft showed comparable strong NIS expression.

### The combined GEM/^131^I^−^ treatment is more effective than either treatment alone

**Figure 3:**
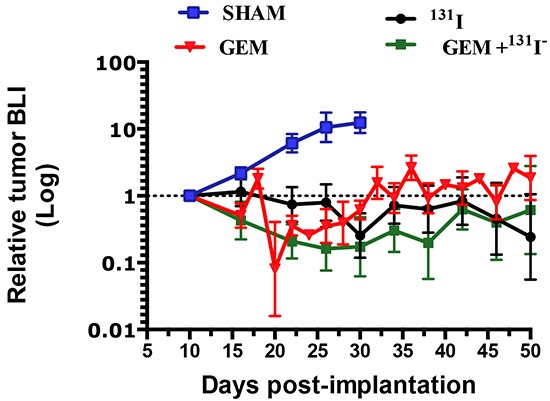
Effects of gemcitabine, ^131^I^−^, and combination treatment on MDA-MB-231Br –NIL MFP tumors MFP xenograft tumors grew over the course of a 50-day period without treatment (Saline, blue solid line), whereas growth was inhibited in tumors treated with GEM (58 mg/Kg x4, red line) or 1 mCi ^131^I^−^ (black line). Dual therapy, GEM 58 mg /kg × 4) plus 1 mCi ^131^I^−^, induced greater tumor regression (green line). Each data point represents the average of results from at least five mice and the corresponding SEM.

In mice bearing orthotopic MFP tumors, five treatments were investigated beginning ten days post-implantation (PI). Exponential tumor growth occurred in untreated mice and all were sacrificed by day 32. Treatment with 1 mCi ^131^I^−^ alone (as a single dose or divided into 2 × 0.5 mCi doses) caused similar growth inhibition with both dosing schemes, as measured by BLI over a 50-day period [Figure 3]. A significant effect of GEM on tumor growth was also observed with 4 doses of 58 mg/kg (174 mg/m^2^) every 3 days. Remarkably, combined treatments of GEM 58 mg/kg × 4 with 1mCi ^131^I^−^ or 2 × 0.5 mCi ^131^I^−^ resulted in the greatest decrease in BLI. No significant weight loss, gait disturbances, or other morbidities were observed with the treatments.

**Figure 4:**
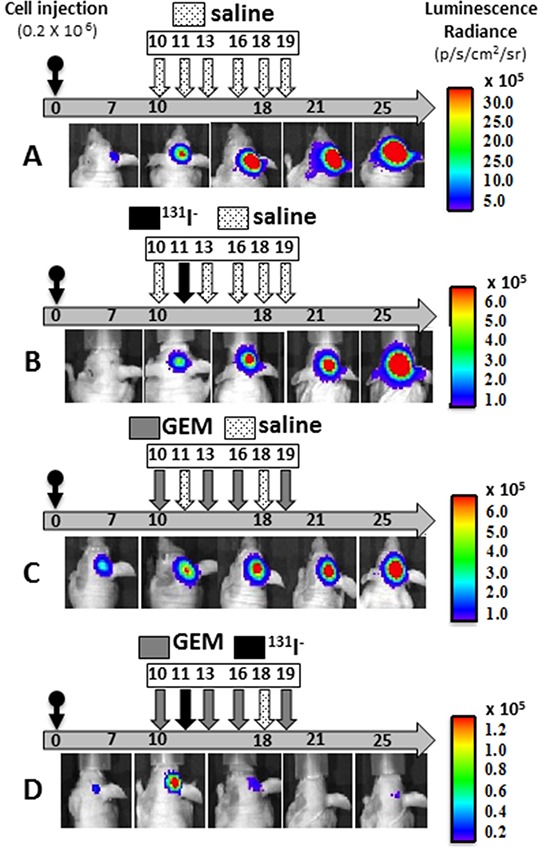
Effects of gemcitabine, ^131^I^−^, and combination treatment on MDA-MB-231Br –NIL BCBM **A–D.** Mice were injected into the right cerebral cortex with 0.2 × 10^6^ cells. Tumor BLI was monitored bi-weekly starting on day 7. Animals with tumors showing exponential growth by day 10 (baseline) were randomly assigned to different treatment groups: [A] saline solution alone (dotted arrows); [B] ^131^I^−^ 1mCi (black arrow) on day 11 and saline other days; [C] GEM 58 mg/Kg (gray arrows) on days 10, 13, 16, 19; [D] dual therapy: GEM on days 10,13,16,19 and ^131^I^−^ (black arrow) on day 11. Pseudo-color BLI images show a representative animal from each group.

**Figure 5:**
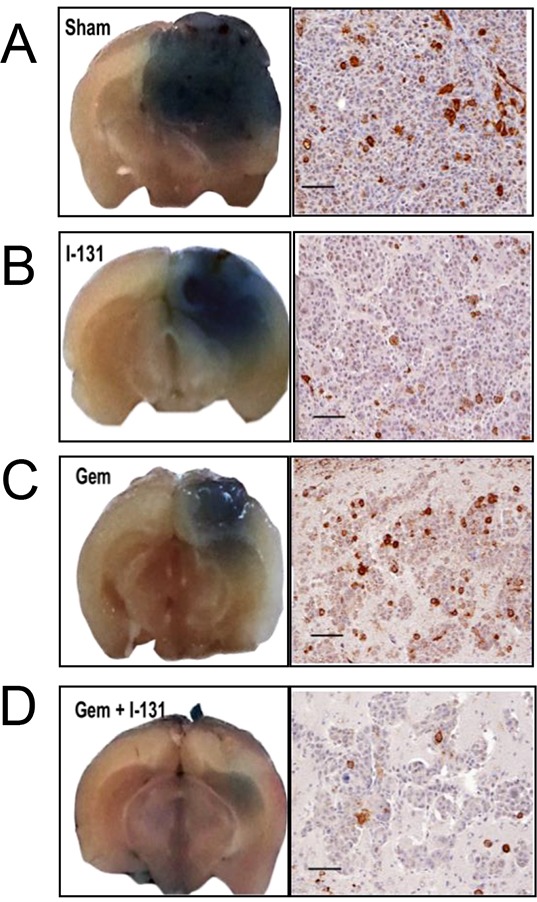
Effectiveness of the dual treatment Representative photographs of whole-brain coronal sections from mice infused with Evans blue prior to sacrifice, highlighting the location of metastases (left). Staining ranged from intensely blue in the larger tumors from sham or saline-treated mice **A.** to smaller ^131^I^−^- and GEM-treated tumors **B.** and **C.** respectively. Dual therapy resulted in the smallest tumors of all **D.** Accompanying panels show histologic sections stained with anti-NIS antibody (scale bars 100 μm). Saline-treated tumors show NIS membrane staining in ~15% of cells, whereas those treated with ^131^I^−^ at least doubled in size, using the BLI criterion, and <5% of their cells were NIS-positive [B]. In GEM-treated tumors *that regressed* [C], the proportion of NIS-positive cells was similar to that in the control group. In the GEM/^131^I^−^ group [D], tumors were smallest or undetectable; in the tumor shown, which was classified as *stable*, <5% of the cells were NIS-positive.

Therapeutic effectiveness was also studied in mice with stereotactically generated BCBMs. A representative example of tumor response for each treatment regimen is depicted in Figure 4A-D. Sham or saline-treated mice had the largest brain tumors, consistent with the BLI measurements and steady growth [Figure 4A and 5A]. The second-largest tumors were those treated with ^131^I^−^ only, indicating limited therapeutic effect [Figure 4B and 5B]. The latter were followed by those treated with GEM alone [Figure 4C and 5C], and the smallest tumors of all occurred in the mice that received dual therapy [Figure 4D and 5D].

NIS expression was remarkably sparse at PID 11, being found in only 5% of tumor cells [Figure 2C]. Later, at PID25, the proportion of NIS-expressing cells in exponentially growing tumors increased, but exhibited regional heterogeneity [Figure 5A]. Specifically, the intracerebral portion of untreated BCBMs exhibited up to 20% NIS-positive cells with noticeably fewer immunoreactive cells in tumor areas extending above the surface of the brain, a finding reminiscent of observations in MFP xenografts.

**Figure 6:**
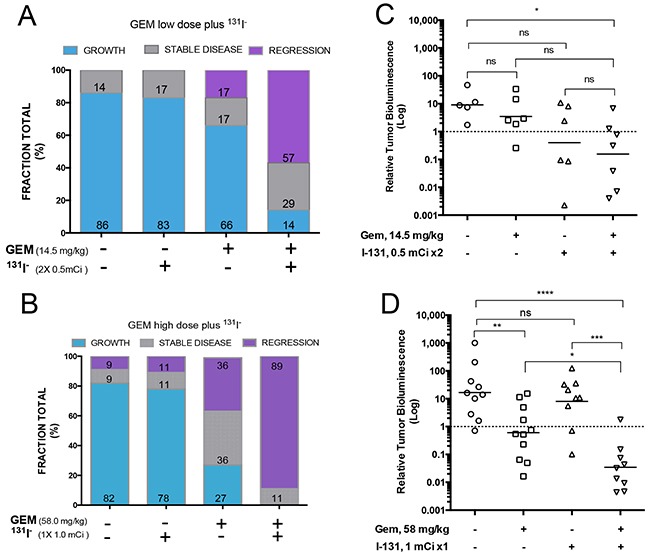
Effects of different treatments on tumor growth **A–B.** Tumor response for each animal was classified as *growth* if BLI was at least twice as intense as the baseline, *regression* if it was half or less as intense, and *stable disease* if BLI remained within the 2-fold parameter on PID25. The percentage of mice in each category is shown in the bar graph. [A] Effects of the low-dose regimens [14.5 mg GEM /kg (days 10, 13, 16, 19) and 2 × 0.5 mCi ^131^I^−^ (days 11 and 18)], with each agent alone or both combined. [B] Effects of the high-dose regimens [GEM 58 mg /kg (days 10, 13, 16, 19) and 1 mCi ^131^I (day 11)], with each agent alone or both combined. **C–D.** Comparison of antitumor activity of low and high dose GEM, ^131^I, and combination treatment on MDA-MB-23-Br-NIL brain tumors at PID25. Treatments and control groups were compared by one way-analysis of variance followed by post hoc comparisons using the Dunnett's multiple-comparisons test. Statistics were performed on log-transformed data. The threshold for significance was set at P<0.05. ns P>0.05, * P≤0.05, ** P≤0.01, *** P≤0.001, **** P≤0.0001.

Tumor response in each mouse was classified using the criterion of a 2-fold change in BLI over the course of treatment through PID25, the timepoint at which treated tumors reached a nadir. BCBMs exhibiting a 2-fold increase in BLI were classified as growing, tumors with a 2-fold decrease as regressing, and all others as exhibiting stable disease. The vast majority of brain metastases in the control group (82-86%) grew [Figure 6A and 6B]. Mice treated with ^131^I^−^ alone showed little or no tumor response: tumors grew in 78-83% of cases, and regression occurred in only 11% of tumors that received a single radioisotope dose [Figure 6B] and 17% of tumors that received divided radioisotope doses [Figure 6A]. By contrast, tumors grew in only 27% of mice under the higher-GEM regimen, whereas 72% of mice exhibited stable disease or tumor regression (36% and 36% respectively) [Figure 6B]. Low-dose GEM alone was associated with growth in 66% of BCBMs animals, and induced far less tumor regression or stability [Figure 6A]. Strikingly, the dual therapy (GEM/^131^I^−^) had a significant effect on BCBMs. No tumors grew under the high-dose combination therapy, only 11% exhibited stable disease, and 89% regressed [Figure 6B]. The low-dose dual therapy further illustrated the synergism between GEM and ^131^I^−^, given the limited effect of each of these drugs alone. The low-dose dual therapy induced tumor regression in 57% of mice, and an additional 29% showed stable disease [Figure 6A]. ANOVA analysis depicts the differences in BLI of surviving mice on PID25 in each of the groups [Figure 6C–6D].

**Figure 7:**
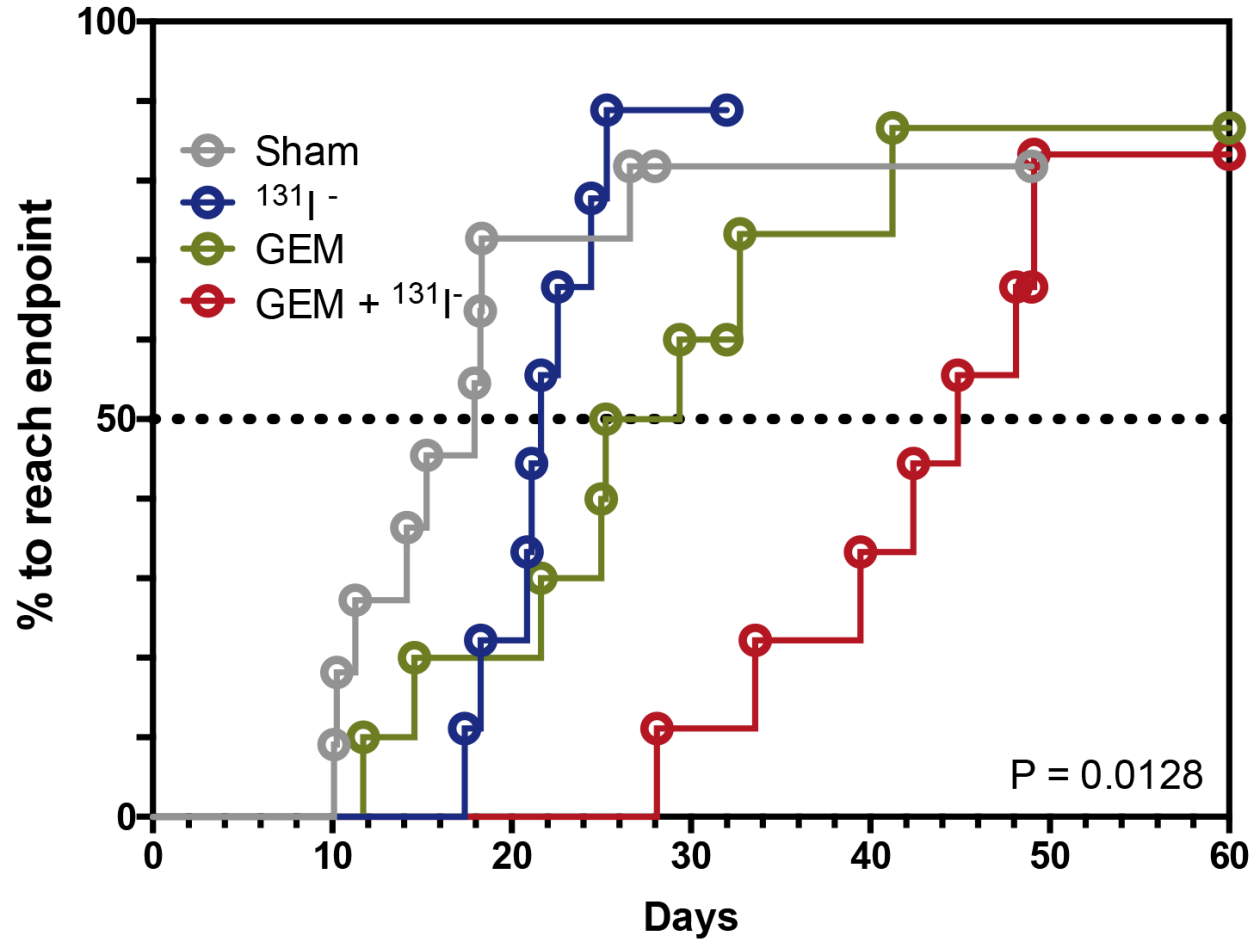
Dual therapy delays tumor progression Kaplan-Meier survival analysis is shown using median survival time for each treatment group or the time required to achieve 5 times the bioluminescence compared to its respective baseline on PID10. Sham or saline-treated [gray], ^131^I^−^ [blue], GEM [green], GEM + ^131^I^−^ [red]. The threshold for significance was set at *p* < 0.05.

A time-to-progression analysis was carried out for mice treated with high-dose GEM [Figure 7]. Time to progression was defined as the time it took for the BLI value to increase 5 times its baseline fluorescence at PID10. Nearly all mice showed some degree of tumor regrowth. The median time to tumor progression was 18 days for untreated, 22 days for ^131^I^−^-treated, 27 days for GEM-treated, and—remarkably—45 days for dual-therapy-treated mice.

## DISCUSSION

Clinically, NIS-mediated radioiodide transport has been the centerpiece of diagnostic tests and ablative interventions in well-differentiated thyroid cancers for over 65 years [5, 25, 26]. ^131^I^−^ treatment is well tolerated, with only a few minor side effects [27, 28]. Therefore, it would be highly desirable to extend this therapeutic approach to NIS-expressing breast cancers [10, 11, 29]. It should be emphasized that the covalent incorporation of I^−^ into thyroglobulin (i.e., I^−^ organification), which occurs in the normal thyroid, is not required for NIS-mediated radioiodide therapy to be effective. This is clearly demonstrated by the fact that NIS-mediated radioiodide therapy is remarkably successful in treating thyroid cancer metastases, even though these metastases lack the microscopic architecture of the thyroid and therefore do not “trap” organified radioiodide the way the healthy thyroid gland does. Moreover, the ablative effect of radioiodide has been reported in multiple preclinical studies of cancers that do not organify I^−^ [30–32].

The well-known bystander effect of ^131^I^−^ (i.e., its ability to ablate non-NIS-expressing cells by being accumulated by surrounding cells) has been demonstrated in NIS gene transfer experiments in various cell lines [31, 33, 34]. ^131^I^−^ has been shown to have a greater ablative effect in cells organized into spheroids than in the same cells grown in monolayers, suggesting that a 3-dimensional arrangement allows cells to be within the field of radiation of other cells that concentrate ^131^I^−^, thereby magnifying the effect of ^131^I^−^[35, 36].

Systemic therapies have decreased the rate of distant metastases in breast cancer and have brought to the fore the daunting challenges of treating brain metastases [1, 37, 38]. One central obstacle in treating BCBMs is that it is difficult for drugs to cross the BBB. Here, we tested a novel strategy combining the radiosensitizing properties of GEM and the radioablative effects of ^131^I^−^ in NIS-expressing breast cancer cells. This strategy was based on the observation that human BCBMs express NIS endogenously: 75% of 28 archival tissues showed NIS protein expression, and 24% evinced focal plasma membrane staining [10]. These striking findings provided the rationale for the translational studies reported here.

BCBMs were generated with a line of braintropic NIS-transduced cells whose proliferation was inhibited *in vitro* by GEM and by ^131^I^−^ [Figure 1D and 1E]. In experimental CNS metastases, tumor size was decreased notably by the combined GEM/^131^I^−^ treatment. This dual therapy elicited a better response than either agent alone, and its effect exceeded the sum of the effects of ^131^I^−^ alone and GEM alone [Figure 6]. The magnitude of the response attests to the radiosensitizing effect of GEM on tumor cells that either actively accumulate ^131^I^−^ (because they express NIS) or are ablated by NIS-mediated ^131^I^−^ accumulation in neighboring cells (i.e., the bystander effect). This point is further illustrated by the results with low GEM dosing. GEM alone caused regression in 17% of tumors and stable disease in an additional 17%, whereas co-administration of GEM and ^131^I^−^ increased the fraction of mice exhibiting tumor regression to 57% (3.3 times greater) [Figure 6A and 6B]. Moreover, time to progression was nearly thrice as long in mice treated with high-dose GEM/^131^I^−^ as in controls [Figure 7].

The bystander effect is likely to have occurred in our BCBM model, given that only ~20% of the BCBMs expressed NIS strongly. In addition, co-administration of a radiosensitizing drug such as GEM sensitized cells to a dose of radiation that alone is not therapeutically effective. Tissue radiosensitivity depends on many factors, including radiation dose, duration of exposure, and the timing of radiation [39]. The low dose GEM approach was based on the clinical practice of reducing the dose of GEM when given concurrently during radiation therapy [40]. For our experiments, a GEM dose that inhibited tumor growth without inducing tumor regression was selected. GEM is a pyrimidine analogue that is phosphorylated intracellularly. Its major metabolite, GEM triphosphate, is competitively incorporated into DNA, impairing DNA synthesis and thereby causing S-phase cell cycle arrest and depletion of the dATP pools [39]. GEM does not significantly bind to plasma proteins; rather, it enters cells by facilitated diffusion. It has been reported that the drug may be preferentially concentrated in brain metastases rather than in normal brain tissue [41]. The effect of GEM is greatest when cells are exposed to relatively low concentrations for at least 24 hours before irradiation, allowing sufficient time for the cells to redistribute into early S-phase. Entry into S-phase and depletion of endogenous dATP pools are critical conditions for radiosensitization of tumor cells. It was for this reason that ^131^I^−^ was administered 24 hours after the first dose of GEM.

Microscopic analysis provided insights into the variability of NIS expression in MFP and brain tumors. Tumors harvested from the MFP 12 days after injection showed strong NIS expression in the centrally located cells and no NIS expression in the more peripheral areas [Figure 2C]. Interestingly, NIS expression in BCBMs showed immunoreactivity in the intracerebral portion of tumors, but sparse immunoreactivity in areas of the same tumors located above the surface of the brain. NIS and luciferase genes are transcribed as a single transcript; therefore, proliferating cell populations in the periphery of a tumor, detectable by increasing BLI, may fail to translate NIS. These preclinical animal models replicate the NIS heterogeneity observed in immunohistochemical studies of human BCBMs, where a relatively small proportion of cells exhibit strong NIS expression [10]. Our results further demonstrate a synergistic effect of low-dose GEM and ^131^I^−^ in MDA-MB-231Br-generated brain metastases. Clinical treatment regimens are characteristically multimodal, often involving a combination of endocrine or cytotoxic agents and external radiation. Synergism between systemic treatments, surgery, and breast irradiation is evidenced in the decreasing local recurrence rates achieved in patients undergoing breast-conserving therapy[42]. NIS protein expression is part of the endogenous repertoire of many TN breast cancers, including BCBMs [Figure 2E] [12]. We showed that this feature is equally well retained in a PDOX [Figure 2D], demonstrating feasibility of potential utility of such a model in future clinical testing. In summary, our findings indicate that the combination of NIS-mediated ^131^I^−^ accumulation and a radiosensitizing drug like GEM may prove an effective treatment for patients with BCBMs.

## MATERIALS AND METHODS

### Cell culture

A braintropic clone of the estrogen, progesterone, and HER2 receptor-negative human breast cancer cell line, MDA-MB-231Br, was generously provided by Dr. Toshiyuki Yoneda (University of Texas Health Science Center at San Antonio) [43]. The cells were not authenticated further. Early passage cells were cultured in RPMI 1640 + L-Glutamine (Gibco; Life Technologies, Inc., Grand Island, NY) supplemented with 10% fetal bovine serum (FBS, Gibco; Life Technologies, Inc., Grand Island, NY) and 1% penicillin-streptomycin (P/S) solution and incubated at 37°C, 5% CO2 atmosphere. Human embryonic kidney fibroblast (HEK 293T) cells were grown in Minimum Essential Medium (MEM, Invitrogen) supplemented with 10% FBS and 1%P/S.

### Cell transduction

MDA-MB-231BR cells were stably transduced with a lentiviral vector containing NIS linked via a bi-cistronic cassette to firefly luciferase 2 (Fluc2) cDNAs, under the CMV promoter (NIS-IRES-LUC= NIL). Briefly, viral particles were produced by transfecting the CS-CMV-NIS-IRES-Fluc plasmid together with defective packaging constructs (pCMV.R8.2) encoding HIV-1 gag, pol, rev and tat and the plasmid (pMD.G) coding for VSVG envelope protein into human embryonic kidney fibroblast (HEK 293T) cells using standard calcium phosphate transfection method as previously described [44]. After transduction, single cell clones were isolated by limiting dilution and characterized for luciferase expression and I^−^ uptake activity.

### In vitro bioluminescence imaging (BLI)

Cells were serially diluted from 100,000 to 25 cells in complete media in 48-well plates and incubated overnight at 37°C. D-Luciferin Firefly potassium salt (Biosynth International Inc, Itasca, IL) was added to each well (150 μg/ml final) 5–10 min before imaging with a Xenogen IVIS-100 imaging system (Caliper Life Sciences, Hopkinton, MA). Exposure time was set to 30-60 sec/plate. Regions of interest (ROI) were drawn around each well and bioluminescent intensity quantified in photons per second using Living Image software (Caliper Life Sciences, Hopkinton, MA).

### In vitro uptake studies

Cell NIS activity was determined with ^99m^ TcO4 at steady-state conditions as described by Weiss *et al*. [45]. In brief, 1 × 10^5^ cells were seeded on 24-well plates and left to attach overnight at 37°C. Uptake was initiated by incubating cells for 1 hour at 37°C with HBSS buffer containing 10 mM Hepes (pH 7.3), 10 μM NaI and 0.1 μCi Na^99m^TcO_4_ per mL. Cells were then washed twice with ice-cold HBSS-Hepes buffer and lysed with RIPA buffer for 20 minutes on ice. Radioactivity of protein lysates was measured in a Cobra II gamma-counter (Packard Cobra, Packard Instrument Inc., Meriden, CT) and normalized to protein content measured by BCA assay (Thermo Scientific-Pierce, Rockford, IL). Each experiment was carried out in triplicate, in parallel with 100 μM KClO_4_ for inhibition of NIS activity.

### Cell proliferation assay

Cells were harvested and seeded at a density of 500 cells/well in 96-well plates, incubated for 24 hours at 37°C, and then GEM (0.0001 to 1000 μM final) was added to each well. After 48 hours, cell viability was measured by the MTS assay according to the manufacturer's recommendations (CellTiter 96AQueous One Solution Reagent, Promega, Madison, WI). Specific absorbance was measured at 490 nm and background at 630 nm with a 96-well plate reader (Biotek power wave XS, Biotek Instrument Inc. Wonooski, VT). Each concentration of GEM was tested in six wells and repeated in at least three independent experiments. The percentage of untreated or control cells surviving, was considered as 100. Percent cell survival was calculated using the following equation: 100* (A490 - A630 treated)/A490 - A630 control).

### Clonogenic cell survival assay

Cells (7 × 10^5^) were seeded on T25 flasks, and allowed to attach overnight. The next day cells were washed twice with warm HBSS and incubated in HBSS supplemented with 10 mM Hepes (pH 7.3), 10 μM Na^131^I containing either 0, 4, 20, 40, 200, or 400 μCi ^131^I^−^ for 7 hours. Following incubation, cells were washed with HBSS-Hepes buffer, harvested, and reseeded at desired densities (2, 5, 10, 20, 50 and 100 cells/cm^2^) in six-well plates. All incubations were done at 37°C. Once colonies developed, cells were fixed with methanol and stained with crystal violet (0.5% w/v methanol). Only colonies containing more than 50 cells were scored. The surviving fractions (SF) were calculated as: (mean plating efficiency of treated cells/mean plating efficiency of control cells)*100%, where the plating efficiency (PE) is the number of colonies divided by the number of cells seeded.

### Animal experiments

Mouse experiments were conducted and approved by the Administrative Panel on Laboratory Animal Care (APLAC) at Stanford University. Six to eight week-old female, athymic, nude mice (Ncr nu/nu) (Taconics, Hudson, NY) were used. Mice were anesthesized for imaging sessions and all procedures carried out with continuous 3% isofluorane (Aerrane, Baxter Healthcare Corp, Deerfield, IL), except for stereotactic tumor cell injections, which were performed using intraperitoneal ketamine/xylazine cocktail (100/10 mg/kg).

To generate mammary fat pad (MFP) xenografts, cells were harvested at 80% confluence, resuspended in HBSS and 1×10^6^ cells injected with an equal volume of Matrigel (BD Biosciences, San Jose, CA). For the brain metastases model, anesthetized mice were secured onto a stereotactic frame (Kopf Instruments, Tujunga, CA). A 0.7-mm burr hole was drilled via a scalp incision overlying the right cerebral hemisphere, 2 mm lateral and 1 mm superior to the bregma using a high-speed micro drill (Fine Science Tools, Foster City, CA). After dural penetration, a 2-μL suspension containing low-passage 2 × 10^5^ MDA-MB-231Br-NIL cells was injected at a depth of 2.5 mm over a four-minute period through a 26-G needle attached to a 10-μL microinjector syringe (Hamilton Co, Reno, NV). The needle was then retracted 0.5 mm/min, the burr hole sealed with bone wax (World Precision Instrument, Inc., Sarasota, FL), and the scalp closed using 5-0 vicryl sutures (Ethicon, Inc, Somerville, NJ).

### In vivo bioluminescence imaging

Tumor growth and response to treatment were assessed longitudinally by in vivo BLI with 150 mg/kg D-Luciferin injected intraperitoneally (i.p). Imaging was initiated within five minutes and sequential images were acquired at 3-minute intervals until peak BLI signal was reached for each animal. In the mice with BCBM, treatment effect was assessed by calculating changes in tumor bioluminescence at each time-point relative to the BLI intensity recorded for that mouse at baseline (PID10).

### In vivo iodide uptake experiments

Two weeks after inoculation, mice bearing MFP tumor xenografts were injected with 40 μCi (1.48 MBq) of Na^123^I in 0.9% saline solution iv. Mice (n = 5) were sacrificed at 2, 4, 6, and 24 hours post-injection. Blood, tumors, and organs were harvested, and radioactivity was counted using a Cobra II gamma-counter (Packard BioScience). Organ uptake was calculated as the percentage of the injected dose per gram of tissue (%ID/g).

### Treatment of mammary fat pad xenografts

Dosing experiments for GEM and ^131^I^−^ were carried out in MFP xenografts. Mice were implanted with methimazole 0.25 mg/21 day release pellet and L-thyroxine 0.1 mg/21-day release pellet (Innovative Research of America, Inc, Sarasota, FL). In addition 10 mg of methimazole were added to 5 ml sterile drinking water and mice were injected with T3 47.6 ng/ 20 μl i.p. daily. GEM hydrochloride (Sagent Pharmaceutical Inc., Schaumburg, IL/Hospira Inc., Lake Forest, IL) was administered 58 mg/kg (174 mg/m^2^) i.p. every 3 days x 4 [46]. ^131^I^−^ therapy was given as 2 doses of 0.5 mCi each 7 days apart (PID11 and PID18).

### Treatment of brain metastases

Two dosing schemes were selected, one replicating the MFP experiments and a second using one-fourth the dose of GEM to minimize drug therapeutic effect. Mice with exponentially growing BCBM on PID10 were randomly assigned to one of four treatment groups (n ≥9 per group): [A] saline sham (0.9% NaCl), [B1] GEM 14.5 mg/Kg or [B2] 58 mg/kg i.p. every 3 days × 4 (days 10, 13, 16 and 19), and [C1] 1mCi ^131^I^−^ on PID11 or [C2] 0.5 mCi ^131^I^−^ on PID11 and 18. For the GEM/^131^I^−^ combination treatments, mice received [D1] 14.5 mg/kg GEM + 2 × 0.5 mCi ^131^I^−^ or [D2] GEM 58 mg/Kg × 4 + 1 mCi ^131^I^−^. Sham and GEM mice received saline instead of ^131^I^−^ while sham and ^131^I^−^ groups received saline instead of GEM. Mice in experiments corresponding to low-dose GEM and divided doses of ^131^I^−^ were sacrificed on PID25.

To suppress thyroidal radioiodide uptake and intrathyroidal organification of I^−^, all mice received daily L-thyroxine (100 μg/kg; Sigma-Aldrich, St. Louis, MO), triiodo-L-thyronine 2.38 μg/kg (Sigma-Aldrich, St. Louis, MO), and methimazole 500 μg/kg (Par Pharmaceutical Inc., Woodcliff Lake, NJ) starting the day of cell inoculation through completion of treatment. Mice underwent BLI imaging twice weekly to assess tumor response and were humanely euthanized if they experienced weight loss in excess of 20% of pre-treatment weight or when moribund. In order to investigate maximal tumor response and time to tumor regrowth or progression, mice were monitored for up to 8 weeks or physical decline, whichever came first. In experiments using low-dose GEM regimens, mice were sacrificed at the time of BLI nadir (PID25). Tumor response was classified as growth, stability, or regression based on a 2-fold change in BLI. The histological appearance and NIS immunohistochemical profile of tumors at this timepoint was analyzed.

### Patient-derived orthotopic xenografts (PDOX)

This procedure was approved by the Stanford University's Research Compliance Office on Human Subjects Research and IRB, as well as Stanford's Administrative Panel on Laboratory Animal Care (APLAC). Informed written consent was obtained from the patient and fresh tumor tissue was taken at the time of surgical excision. The sample was placed in ice cold RPMI 1640 medium supplemented with penicillin/streptomycin and 10% heat-inactivated FBS (Invitrogen-Life Technologies, Carlsbad, CA, USA) as previously described by our group [47]. One to two mm fragments were mixed with LDEV-free Matrigel (BD Biosciences, San Jose, CA, USA) and orthotopically implanted into the MFPs of 5 female NOD SCID mice (NOD. CB17-*Prkdc^scid^*/J, Jackson Laboratory West, Sacramento, CA, USA). Mice were housed in a pathogen-free animal environment. Once established, PDOX tumors were expanded by passaging from mouse to mouse. After a second passage, one PDOX tumor was fixed in formalin and embedded in paraffin for immunohistochemical studies.

### Immunohistochemistry

Five-micron formalin-fixed, paraffin-embedded tissue sections were deparaffinized, rehydrated through graded alcohol changes and then subjected to heat-induced epitope retrieval in 0.01 M citrate buffer (pH 6.0) for 30 minutes at a sub-boiling temperature. Immunohistochemistry (IHC) staining was carried with biotin-free catalyzed signal amplification kit (CSA II; DAKO, Carpinteria, CA). Slides were counterstained with hematoxylin before mounting. Appropriate positive (thyroid Graves' disease) and negative (no primary antibody) controls were included with each IHC run. NIS expression was determined using a custom affinity-purified polyclonal anti-human NIS antibody (AnaSpec, Fremont, CA) generated against the last 13 amino acids of the carboxy-terminal end of the protein.

### Data analysis

Statistics were performed using GraphPad Prism version 6.00 for Mac OSX (GraphPad Software, La Jolla California USA).
